# Webcam use in German neonatological intensive care units: an interview study on parental expectations and experiences

**DOI:** 10.1186/s12913-021-06901-7

**Published:** 2021-09-15

**Authors:** Alinda Reimer, Laura Mause, Jan Hoffmann, Pauline Mantell, Johanne Stümpel, Till Dresbach, Nadine Scholten

**Affiliations:** 1 University of Cologne, Faculty of Medicine and University Hospital Cologne, Faculty of Human Sciences, Institute for Medical Sociology, Health Services Research, and Rehabilitation Science (IMVR), Eupener Str. 129, 50933 Cologne, Germany; 2 University Hospital Cologne, Research Unit Ethics, Universitätsstr. 91, 50931 Cologne, Germany; 3 Cologne Center for Ethics, Rights, Economics, and Social Sciences of Health (CERES), University of Cologne, Universitätsstraße 91, 50931 Cologne, Germany; 4 Department of Neonatology and Pediatric Intensive Care Medicine, University Hospital Bonn, Venusberg-Campus 1, 53127 Bonn, Germany

**Keywords:** Webcam, Video, Virtual visitation, Parents, NICU

## Abstract

**Background:**

To bridge the physical distance between parents and children during a neonatal intensive care unit (NICU) stay, webcams are used in few German NICUs. They allow parents to view their infant even when they cannot be present on the ward. The aim of the study was to explore the factors for and against webcam use that parents with or without webcam use encountered.

**Methods:**

Guideline-based, semi-structured qualitative interviews were conducted in the period from September 2019 to August 2020. Interview transcripts were analysed using a category-based content analysis. The categories were generated in a combined deductive–inductive procedure.

**Results:**

We interviewed 33 mothers and seven fathers. Parents with webcam experience emphasised positive aspects concerning their webcam use. Factors that increased webcam acceptance included feeling certain about the child’s well-being and an increased sense of proximity. Only a few critical voices emerged from parents who had webcam experience, e.g. regarding privacy concerns.

Parents who had no experience with webcam use showed ambivalence. On the one hand, they expressed a positive attitude towards the webcam system and acknowledged that webcam use could result in feelings of control. On the other hand, reservations emerged concerning an increase of mental stress or a negative influence on parental visitation behaviour.

**Conclusion:**

In addition to the parents’ positive experiences with webcam use, results show a need within parents who lacked webcam experience. Despite some criticism, it was evident that webcam use was primarily seen as an opportunity to counteract the negative consequences of separation in the postnatal phase.

**Trial registration:**

The Neo-CamCare study is registered at the German Clinical Trials Register.

DRKS-ID: DRKS00017755.

Date of Registration in DRKS: 25-09-2019.

## Background

Several studies have focused on the challenges of a preterm birth for parental well-being. These challenges include parental distress, poor sleep, increased risk of ill health and the development of negative feelings towards one’s infant [1–3]. Preterm births also involve a risk of reduced emotional attachment [4] and impaired confidence in the parental role [5, 6]. Parents have reported feelings of existential loneliness and have described negative effects on their transition to parenthood [7]. These negative consequences originate partly from a separation of parent and child [8].

Because of their unstable health, including neonates with very low birthweight (VLBW), who are born weighing less than 1500 g, are transferred to a neonatological intensive care unit (NICU) for observation and treatment. In German NICUs, usually it is not possible for parents to remain at their child’s bedside throughout their hospitalisation. This can be due to various reasons depending on the NICU’s structural possibilities (e.g. lack of space) or on the parents’ individual situations (e.g. other family obligations). In addition, many clinics restrict visitation by parents. These restrictions often became more stringent during the SARS-CoV-2 pandemic, depending on the federal states’ requirements and local conditions. Hence, the postnatal period is associated with a prolonged separation of parent and child. This brings parental feelings of helplessness and loss of control to the fore [9, 10].

Implementing webcam technology in the NICU setting represents a possibility to overcome the separation’s negative effects on parental well-being. Webcams allow parents to view their critically ill and premature newborns, including neonates with very low birthweight (VLBW), on a monitor when they cannot be present in the ward. A password-protected system enables virtual visitation through a web-enabled device with an encrypted connection.

Webcam use is not widespread internationally and relatively few NICUs have previously used webcams. However, several studies have considered the effects this technology has on parental well-being.

Qualitative studies have shown that parents generally express a positive attitude towards webcams in the NICU setting: Aspects emphasised are a perceived improvement of physical and emotional well-being and involvement in the social environment [11]. Feelings of closeness and intensified bonding between parent and infant are underscored [11, 12]. Furthermore, it has been shown that webcams are viewed as helpful, as long as certain measures – such as data protection and assurance of informed consent – are followed [12]. A study from Ireland by Hawkes et al. [13] outlined that parents believed to experience less stress and feelings of guilt when offered a webcam. A prospective, observational study by Weber et al. [14], which compared parents with and without webcam access in the United States, elicited that parents who used webcams showed a tendency to perceive more involvement in the neonate’s care. Previous research has also identified certain challenges posed by webcam technology. Parents expressed feelings of anxiety due to the webcam’s omnipresence [11]. Also, a mixed methods study from the United States referred to increased feelings of stress and anxiety in few parents. These feelings could arise in situations when watching one’s child in discomfort and not being physically present [15]. Other reasons against the use of webcams were of cultural or economic origin [16].

A growing body of literature indicates the potential benefits of webcam use in the NICU setting. However, parental experiences with a webcam system have not been investigated with regard to expectations about the webcam use. Previous studies have mainly focused on the views of parents who had actually used a webcam system during their child’s NICU stay. In addition to these actual experiences concerning webcam use in the NICU setting, it is important to consider the expectations of parents who lack such experience. Understanding parental expectations for a webcam system and exploring their actual experiences are necessary tasks to investigate the need for webcams during an NICU stay, and to assess the technology’s consequences.

Therefore, our study identified and compared parental expectations with parents’ actual experiences, using the following research question: Which factors for and against webcam use can be identified from the perspective of parents without webcam experience in contrast to parents with webcam experience?

## Methods

### Study design

The Neo-CamCare project [17] was initiated to evaluate webcam use in German NICUs for the first time. This project surveys the parents’ and health care professionals’ perspectives on webcam use. For the parents’ evaluation a randomised controlled study in a waiting group control design is conducted in four different NICUs throughout Germany. Parents participating spend one month each with and without a webcam. As infants with a VLBW require long-term care in the NICU and are more likely to stay hospitalised for the observation period, these were included in the Neo-CamCare study. Therefore, the birthweight was also an inclusion criterion that was used for participant selection in this present paper.

Part of the study covers the parents’ perspectives and perceptions about webcam implementation in NICUs. The present paper contributes to that part of the project.

The present paper contributes to that part of the project and focuses on devices that do not support communication webcam but merely transmit the child's image. Therefore, health data, e.g. through vital signs monitoring devices, is not transferred. Webcams can be deactivated or repositioned in case of nursing procedures or medical emergencies. Except for these circumstances, webcam-use in participating clinics is not limited to specific visiting hours, so parents can view their child around the clock.

To gain deeper understanding of the topic and discover new areas for research, a qualitative approach was chosen. To answer the research question, semi-structured interviews were conducted with parents of VLBW infants. Participants gave their written informed consent prior to participation by post or in person, and were granted a financial allowance of EUR 50,00. Before the interviews, participants were informed about the research goals and were asked to answer freely. A pseudonymised evaluation and strictly confidential handling of their data were guaranteed. The study was approved by the ethical review committee of the Medical Faculty of the University of Cologne, in consultation with the ethics committee of the Medical Faculty of the University of Bonn (number 19-1232).

### Participant selection and data collection

Two research teams conducted the interviews independently using the same interview guide (Table 1). The use of two teams allowed for investigator triangulation and collaborative coding. The teams consisted of researchers from the Institute of Medical Sociology, Health Services Research and Rehabilitation Science (IMVR) at the University of Cologne and the Research Unit Ethics at the University of Cologne. The data represent the findings from both research teams. The teams followed the methodological approach described here.

**Table 1:** Aspects covered in interview guide

1. Pregnancy and admission to the NICU	
2. Experiences at the NICU	
3. Experiences after the child’s discharge (if applicable)	
**4. Perceptions and expectations about webcam use in the NICU setting**	
◦ What do you associate with webcam use in the NICU?	
◦ What is your attitude towards webcam use in the NICU?	
◦ Do you see/expect advantages concerning webcams in the NICU setting? Where do you see those?	
◦ Do you see/expect disadvantages concerning webcams in the NICU setting? Where do you see those? / What kinds of fears and concerns do you associate with webcam use?	
◦ Do the webcams have an influence on breast milk expression? Do you expect the webcams to have an influence on breast milk expression?	
◦ If you were to decide, would you use the webcam technology (again)? What are the reasons for your decision?	
◦ What should be considered when using webcams in the NICU setting?	
5. Attitude towards progress in technology and data protection	

The lead author, AR (Research Associate, Health Services Researcher, M.Sc.), together with CJ (Research Associate, Medical Manager, M.A), PM (Research Associate, Health Economist, Dipl. Ing.) and JS (Research Associate, Health Economist, M.Sc.) conducted all interviews. These female researchers are all experts who have previously worked in the qualitative paradigm.

The aim was to obtain a sample which was as diverse as possible in terms of parental socio-economic status, parental age, the child’s hospitalisation and birth weight. Participants were recruited purposively by a study nurse, via social media, and personal contacts. We stratified this sample by whether or not parents have used webcams to ensure equal representation according to these dimensions. Parents were acquired from various NICUs around Germany. Not every NICU offered a webcam system at the time of the interviews. Therefore, not all off the parents had an option to use a webcam. Parents without webcam experience were either accommodated in NICUs that did not offer the webcam technology or decided not to use one. All participants invited for interviews agreed to participate and there were no dropouts.

To meet the inclusion criteria, the participants’ children were required to have a birthweight of less than 1500 g. It did not matter whether the child was still hospitalised or had already been discharged. Parents with discharged children were interviewed on average 16.4 months after discharge. Parents whose children had died or who were not hospitalised in German NICUs were not considered for an interview. Interviews were conducted over a seven-month-period from September 2019 to August 2020.

Interviews took place where the parents found it most convenient, which was either in their domestic environment, a parents’ room in the hospital or at the IMVR in Cologne, Germany. Some parents wished to be accompanied by their partner; hence, some conversations were undertaken as paired interviews with both parents present. The SARS-CoV-2 pandemic complicated the interviews. Therefore, virtual or telephonic interviews were offered as an alternative to face-to-face-meetings.

Each interview started after a personal introduction and explanation of the research interest. Hence, a trustworthy and comfortable atmosphere between researchers and participants was established. Parents were asked about their own and their children’s demographic data (Table 2) after the conduction of interviews. The interviews followed the guide shown in Table 1, which was adjusted depending on whether the parents being interviewed had webcam experience. The guide was piloted before the interviews were initiated. Interviews were conducted until data saturation was reached.

**Table 2:** Sample and interview data

***Participants****(N = 40)*	***Frequency***
***Participant’s sex***	
*male*	*7*
*female*	*33*
***Participant’s age***	
*20–25 years*	*3*
*26–30 years*	*7*
*31–35 years*	*14*
*36–40 years*	*12*
*41–45 years*	*3*
*46–50 years*	*1*
***Educational background***	
*primary education*	*1*
*secondary education*	*14*
*higher education*	*21*
*no information*	*4*
***Marital or relationship status***	
*married or in a relationship*	*38*
*single or separated*	*2*
***Number of children****(other than the preterm infant)*	
*0*	*26*
*1*	*9*
*2*	*4*
*3*	*1*
***Multiple birth*** (twins or more)	
*yes*	*10*
*no*	*30*
***Child’s birthweight****(in grams)*^***a***^	
*< 500*	*6*
*500–750*	*22*
*751–1000*	*5*
*1001–1250*	*6*
*1251–1500*	*9*
***Child’s hospitalisation status at time of interview***	
*parents with children hospitalised*	*15*
*parents with discharged children*	*25*
***Webcam use during NICU stay***	
*yes*	*22*
*no*	*18*
***Type of interview***	
*virtual* via *video communication*	*11*
*telephonic*	*9*
*private and face-to-face*	*20*

^a^The number of children exceeds the number of interviews because for twins, both children were considered

Interviews were audio-recorded and transcribed verbatim. Names, locations and potentially identifying details were removed. If necessary, interviewers took field notes after the conduction of interviews to supplement audio-recording. There was no necessity to repeat any interviews. The transcripts were checked for accuracy by the lead author and an undergraduate study assistant.

### Data analysis

Interview transcripts were analysed thematically by applying a qualitative content analysis, based on Kuckartz [18]. This systematic approach enables a content-oriented evaluation by structuring the data into categories and subcategories. We chose this method to explore the variety of parental viewpoints and to provide a structured overview of the interview material. The analysis focused on interview content concerning factors for and against webcam use, which was addressed by parents with actual webcam experience and those who lacked such experience.

The thematic content analysis was based on the transcripts and was performed in a combined deductive–inductive approach. MAXQDA (version 2020) was the tool used for the qualitative coding process. Factors for and against webcam use were analysed and differentiated between parents with and without webcam experience concerning.

The analysis was performed by five researchers (AR, JH, LM, PM and JS). Transcript data regarding parental perceptions and expectations about webcam use (Table 1) were included. The analysis identified the commonalities and differences between the two parental groups.

The category-based evaluation consisted of a multi-stage process of generating categories and coding text segments. First, all analysts coded the data deductively, based on the research question, independently of each other. Categories were deductively determined based on factors for and against webcam use. Then, new codes were created inductively within these categories. The data were revised and reviewed by the analysts. After coding part of the material, the analysists conducted credibility checks to constantly check for accuracy in the coding. Discrepancies in coding were revised where necessary. Codes were then sorted into main and sub-themes, which built up a coding frame. The themes were tested independently and reviewed until the analysts agreed upon a frame that allowed the whole transcript material to be categorized systematically. The final coding frame is illustrated in Table 3.

**Table 3:** Categories of content analysis

1. **PRO: Factors for webcam use in the NICU setting**	
1.1. Feelings of control and reassurance	
1.2. Increased feeling of proximity	
1.3. Positive influence on breast milk expression	
1.4. Improved relationship between parents and health care professionals	
2. **CONTRA: Factors against webcam use in the NICU setting**	
2.1. Increase in mental stress	
2.1.1. *Urge for control*	
2.1.2. *Reinforcement of fears*	
2.1.3. *Feelings of powerlessness*	
2.2. Negative influence on parental visitation behaviour	
2.3. Impaired relationship between parents and health care professionals	
2.4. Privacy concerns	

Despite the independent evaluation by two teams, the researchers developed consistent category systems with identical themes. This consistency confirms theoretical saturation and a high intercoder reliability.

To ensure a quality report, the researchers were guided by the “Consolidated criteria for reporting qualitative research (COREQ): A 32-item checklist for interviews and focus groups” [19].

## Results

### Participant characteristics

In total, 33 mothers and seven fathers (*n* = 40) were interviewed. Of those, 36 interviews were conducted with individual parents, two paired couples of mothers and fathers were interviewed together. On average, each interview lasted about 46 min (min. 28 min; max. 94 min). Of the 15 parents with hospitalised children, eight have used a webcam. The mean corrected age of discharged children at the time of the interview was 17,7 months**.** Further information concerning the sample characteristics and interviews is shown in Table 2.

### Findings

The main themes ‘*factors for webcam use in the NICU setting’* and ‘*factors against webcam use in the NICU setting’* emerged during the analysis. The content related to these themes is presented below. Findings are illustrated for each factor by first describing the perception of parents who lacked webcam experience; thereafter, the experiences of parents who had used a webcam system are presented. Table 3 provides an overview of the themes mentioned by parents with and without webcam experience.

### Factors for webcam use in the NICU setting: experiences and expectations

Of the parents interviewed, 34 stated that they chose (*n* = 23) or would have chosen to use a webcam (*n* = 11) if they had the choice. The value of the webcam was mostly recognized as overweighing the possible disadvantages. In terms of beneficial aspects, the following themes emerged: feelings of control and reassurance; increased feeling of proximity; positive influence on breast milk expression; improved relationship between parents and health care professionals.

#### Feelings of control and reassurance

Parents with and without webcam experience reported that reassurance about their child’s well-being was a highly relevant aspect regarding webcam use. Below, perspectives associated with increased control and reassurance concerning parents’ expectations and actual experiences are explained.

The thought of the possibility to view the child’s livestream was appraised as a gain in control by parents without webcam experience. When not present at the ward, parents frequently perceived information about their child to lack transparency. Therefore, parents expressed a desire to reduce this information gap, viewing the webcam as a possible solution. They reported that an increase in control was vital to establish a greater sense of self-efficacy. In addition, the livestream was believed to promote relaxation, as parents reported their mental stress to decrease when they were certain about the child’s well-being. Another key benefit supporting this feeling of reassurance was observing the child after leaving the ward. This was expected to make it less stressful to return home. The livestream also enabled finding rest during a tense period. The livestream’s calming effect was expected to occur in situations of parental restlessness or worry or at times of needing assurance (e.g. when going to bed or waking up at night). This calming effect was associated with better sleep, along with improved mental balance. Therefore, webcams were also anticipated to maximise leisure time outside of the hospital as parents could be informed consistently about the child’s condition.

The views of parents who had used a webcam system corresponded to the above perceptions. They viewed the technology as a support to reduce their inner restlessness and to help them relax despite the stressful situation. Webcam use also reportedly decreased feelings of guilt when not present on the ward around the clock.

*“I think it’s good for the children if the parents are a bit more relaxed: [The webcam] leads to better sleep within parents or allows them to have breakfast prior to their hospital visit, because they already had a look [at their child in the morning] and just arrive [at the NICU] with a bit more energy. Then they will benefit somehow.” (Interview no. 13: Mother without webcam experience)*

*“Fortunately, we had the chance to use the webcam. I always woke up at night because I was so scared something might have happened. Then I always checked [my child via webcam]. That gave me a bit of security again.” (Interview no. 34: Mother with webcam experience)*

#### Increased feeling of proximity

Webcam use was associated with an increased sense of proximity with one’s child. According to the parental views, this was established by enhancing a feeling of emotional closeness and by overcoming the spatial distance incurred by the NICU stay. Parents who lived further away, had other children to care for, or who had to work shortly after the birth, appeared to have more difficulties visiting the NICU. They emphasised the significance of a webcam due to their lack of opportunities to see their child in person. Most parents agreed that webcam use was an advantage under such circumstances. This point was especially highlighted by parents without webcam experience, who described various situations in which they felt the need to reduce the physical distance between them and their children. Hence, viewing the livestream was seen as a possibility to reduce the longing for the child by parents who lacked webcam experience.

A main factor associated with increased feelings of closeness was the thought of experiencing important moments (e.g. the child’s first bath or a physiotherapy session) in the child’s life. The parents would otherwise not have been a part of these if not present on the ward.

The livestream helped parents with webcam experience to feel more connected to their children, despite the distance the NICU stay entailed. When hospitalisation had complicated the bonding process, the webcam functioned as a substitute for personal visits when these were unfeasible. In addition, the technology was considered valuable if parents lacked the opportunity to see their child on the ward – for example, because of a parent’s own inpatient treatment. This aspect was mentioned by both parental groups.

Webcams were also viewed advantageous because they enabled sharing one’s child’s livestream with relatives and friends. According to parents in both groups, visiting restrictions or distance from the ward were barriers to a close social environment to see their child. The technology offered a way to overcome these obstacles and granted a possibility to show the child to other people.

*“When I had to be at work, I always looked at [my child through the livestream]. You are just calmer. […] Although you cannot be [with your child], you can have a look at it.” (Interview no. 8: Father with webcam experience)*

*“I think [the webcam] could be [essential] for parents who have more children, live further away or are not in a position to visit for health reasons. It would make it much easier for them to develop a bond with their child.” (Interview no. 13: Mother without webcam experience)*

#### Positive influence on breast milk expression

Most of the mothers who were interviewed described difficulties in expressing their breast milk. Mothers without webcam experience partly believed that viewing the livestream would have had a positive influence on breast milk expression, as they expected to have an emotional response to the child’s moving image. This was also assumed to be more helpful than being supported by a photo or recorded video of their child.

The increase in breast milk – or the ability to express it – was an advantage that was similarly highlighted by most mothers with webcam experience. These mothers described the ability to express breast milk more often and in larger volumes when watching their child through the webcam. Webcam use reinforced a feeling of intimacy and emotional well-being, which seemed to stimulate the expression of breast milk. Notably, mothers who had limited mobility after the birth or who were hospitalised themselves believed in the webcam’s positive influence on breast milk expression.

*“I think, it would have been a completely different thing if [I] could have really watched the baby* via *livestream while pumping the milk [...] I can imagine that it really generates a completely different emotion and really makes it easier for you when you’re sitting on the sofa at home. (Interview no. 14: Mother without webcam experience)*

*“The webcam helped to promote [breast milk expression]. I have noticed this. Although I didn’t really have any problems with breast milk expression myself, I noticed that looking at my child had a positive effect on it.” (Interview no. 29: Mother with webcam experience)*

#### Improved relationship between parents and health care professionals

In some instances, parents without webcam experience believed that health care professionals felt greater responsibility when webcams were present than when they were not, because parents might notice any carelessness. This would lead to increased vigilance among NICU staff and would increase the parents’ confidence in the hospital care.

Parents with webcam experience emphasised their ability to obtain information about their child independently from the medical staff by viewing the livestream. Similarly, webcams were described as a tool to support communication between health care professionals and parents. The livestream enabled parents to assist health care professionals by informing them of any discomfort the child might experience, such as a displaced high-flow mask. This virtual involvement in care activities was accompanied by the assumption that health care professionals have less work. Parents received visual information via the webcam that they would normally obtain from health care professionals – such as by phone calls. Parents with webcam experience also reported that the caregivers had strived to make them comfortable through the webcam access, thereby reinforcing the parental trust.

*“We were very happy to see [that our child was taken care of]. We were always thrilled to observe that the nurses really tried to create a situation as if we were there.” (Interview no. 34: Mother with webcam experience)*

*“In situations where I simply could not [visit my child], I would have found it quite nice to just […] take a look at it. I think I would have been happier at that moment. […] this way I would not have to interrupt the nurses in their work routines […].” (Interview no. 13: Mother without webcam experience)*

### Factors against webcam use in the NICU setting: experiences and expectations

Several major themes regarding factors against webcam use were specified. These were an increase in mental stress; negative influence on parental visitation behaviour; impaired relationship between parents and health care professionals; and privacy concerns.

#### Increase in mental stress

An increase in parental mental stress was a crucial aspect that was raised, especially by parents who had not used a webcam system. An increase in anxiety ascribed to webcams originated from several causes, with the following three sub-themes emerging:

##### Urge for control

Several parents emphasised that webcams could reinforce their urge to constantly control their child’s condition. Webcam devices were said to satisfy the need to view their child but also triggered inner tension. The technology was believed to enhance the feeling of pressure to watch one’s child; hence, webcam use was perceived as potentially stressful by parents in both groups. The webcam’s omnipresence and knowing that the livestream was constantly available would make it challenging to find any rest. Parents without webcam experience generally believed that this constant availability of their child’s live image could jeopardize their free time. Therefore, the impact of webcam use was considered a possible stress factor because all the parents’ time resources would be used for webcam use. This point was also associated with an anticipated lack of sleep; parents were worried that they might wake up at night, desiring to watch their child.

The urge for control was accompanied by the concern that relatives – who also felt the need to control the child’s well-being – would bother parents if they had webcam access. Some parents without webcam experience assumed that relatives watching the livestream might dramatize the situations observed. Hence, they might unnecessarily raise concerns if they viewed the child in alleged discomfort.

Parents with actual webcam experience seldom reported an urge for control associated with the presence of a webcam. However, one mother stated that webcam use reinforced her urge to watch her child during every available minute. Hence, her livestream use generated some mental stress. Apart from that, this aspect was of minor relevance for parents with webcam experience.

*“I couldn’t focus on other things because I had the webcam, because I always watched my cell phone […] in order to see the child, no matter what I was doing.” (Interview no. 11: Mother with webcam experience)*

*“If I had known that I only had to press a button to see my daughter, I would have looked at the screen all the time. That would have stressed me out too much.” (Interview no. 9: Mother without webcam experience)*

##### Reinforcement of fears

Another concern raised was worry about enhanced parental anxiety due to the technology. As webcams are switched to standby mode during routine medical treatment, or are forgotten to be realigned afterwards, the livestream might not be available continuously. Parents without webcam experience feared the livestream’s inaccessibility; this prospect evoked thoughts about possible danger to the child and increased their anxiety. The inaccessibility of livestream seemed particularly stressful outside routine hours for medical treatment, as emergency procedures were thought to be more likely.

Additionally, the fear of viewing unpleasant situations through the livestream was articulated. Parents without webcam experience were afraid to observe their child’s discomfort through webcam use. Moreover, the perception of such conditions could imply that the child was being clinically neglected. Therefore, webcam devices were perceived as being an additional burden. Similarly, a few parents with webcam experience reported feeling anxious when they experienced an inaccessible livestream.

*“[*Although the doctors explained to us that the webcam would be inactive at times,*]* it was a strange feeling because you didn’t know if something has happened or the caretakers forgot to turn the webcam back on.” (Interview no. 30: Mother with webcam experience).

*“One is always afraid that the worst will happen [...] maybe at the time [when the webcam is turned off] an ophthalmologist or a physiotherapist merely has to examine your child routinely [...] and the webcam will be turned off. I would have developed a real panic if it would be turned off [unexpectedly].” (Interview no. 17: Mother without webcam experience)*

##### Feelings of powerlessness

Parents are usually not capable of intervening when viewing their child’s discomfort via livestream. Hence, webcam use was described to evoke some feelings of powerlessness. Especially among parents who lacked webcam experience, such thoughts evoked strong emotional discomfort. A few parents with webcam experience confirmed such sentiments. These feelings of powerlessness were generally stronger for parents with longer distances to travel to reach the ward; such parents were perceived to be helplessly exposed to view their child’s discomfort. Their long journey to the ward would make it difficult to intervene if they saw an emergency.

*“[In situations where I saw my child crying], I wanted to get in the car and drive [to the hospital] to help, to calm her down.” (Interview no. 10: Mother with webcam experience)*

*“I think [the livestream] would totally fear me, because I would worry all the time, ‘what is it [with my child] exactly?’ And I think it would also worry me if I saw the [livestream] when I just arrived home and I imagine that I suddenly see that my son is having a total tantrum and I am not with him.” (Interview no. 19: Mother without webcam experience)*

#### Negative influence on parental visitation behaviour

Another issue raised by parents without webcam experience was the risk of a negative influence on parental visitation behaviour. The constant availability of the livestream was feared to substitute for personal visits to the NICU or to reduce their duration. Parents whose visits tended to be brief were assumed to be at risk of missing their visits more often or of shortening their duration even more, because the webcam would enable digital visitation.

This issue was partly linked to the relevance of physical proximity to one’s child. If webcam use led to less parental presence, it was believed to endanger the affective bond between parents and their children. However, the topic of visitation behaviour was not addressed by parents with actual webcam experience.

*“[When I was using the webcam,] I was present [at my child’s bedside] as much as I was [without it].” (Interview no. 11: Mother with webcam experience)*

*“I’m just a bit sceptical, because I don’t know if I would spend a few hours less [with my daughter], although it’s actually important for her that I am present, because she doesn’t really have any advantage from the webcam, no. So, whether there is a screen or another thing hanging over her, she consciously does not notice. But she senses whether I’m with her or not. I sometimes think, that I maybe would be a bit less on the spot. [The webcam] might not have been so good for her.” (Interview no. 13: Mother without webcam experience)*

#### Impaired relationship between parents and health care professionals

Another possible challenge was mainly addressed by parents without webcam experience. This was the webcam’s potential negative impact on the relationship between health care professionals and parents. As described above, parents’ impressions of clinical work could be negatively influenced when observing one’s child in discomfort via livestream. Some parents thought that health care professionals might be accused of negligent behaviour if parents noticed a prolonged absence, and such incidents could lead to recrimination. Thus, webcams could generate mistrust and increase the need for parental control. The technology was thus deemed to place some strain on the relationship between health care professionals and parents.

An increase in parental calls due to monitoring through the webcam was another worry expressed. Webcam use was assumed to increase parental enquiries, resulting in additional stress for health care professionals. However, few parents with actual webcam experience reported that webcam-related tasks seemed to bother the clinical staff. Parents reported that health care professionals rarely seemed strained by requests to align the webcam or to turn it back on.

Furthermore, parents reported inhibitions to call NICU staff too often. However, their reluctance to call the staff was associated with feelings of uneasiness, since they still had concerns about the well-being of the child.

*“I can’t really report anything negative about [the webcam’s functioning], apart from situations when [caretakers] forget to realign the webcam [...].” (Interview no. 21, Mother with webcam experience)*

*“And then I could also imagine that it’s really annoying for the nurses, when there’s another mom calling every ten minutes [...] saying ‘The pacifier fell out. Can you go see my child?’ That must drive you crazy.” (Interview no. 9, Mother without webcam experience)*

#### Privacy concerns

Parental and child privacy was also an aspect underscored in the interviews. Data protection plays a role in this context, regarding the use of social media and new technologies in general but especially with webcam use. Critical views were expressed by parents without webcam experience, especially those who dealt with data protection professionally. These parents stated that it was essential to provide completely secured encryption. Moreover, transparency about the servers’ locations and secure data centres were stated as requirements for proper implementation.

Parents with webcam experience described their misgivings about the fact that parents and their relatives could constantly watch the child’s image. The external appearance of premature infants, which often differs markedly from the prevailing image of full-term infants, and the sometimes clearly visible vulnerability (e.g. through tubes and wiring) was also mentioned in this context. The way in which the livestream was accessible to the family environment varied greatly among the parents surveyed.

*“I find it very important that not everyone has access to my child’s data and that the webcam’s focus is only on the child, without showing a monitor and other information.” (Interview no. 10: Mother with webcam experience)*

*“Well, it’s got to be completely encrypted. [...] what servers would it run on? Where does [the data] go? Does it go to the States? Does it stay in Germany, does it stay in the European Union?” (Interview no. 23: Father without webcam experience)*

## Discussion

This study’s objective was to qualitatively identify factors for and against webcam use from a parental point of view. The perspectives of parents who either had or lacked webcam experience were probed, to compare the parental expectations and the actual experiences concerning webcam use. In order to contrast these parental perspectives in the current research, the derived themes are discussed in this section.

A major finding to emerge from the analysis was the discrepancy between parental expectations and experiences. Fears and worries expressed by parents without webcam experience were seldom mentioned by parents who had actually used webcams. Therefore, the findings suggest that negative expectations generally did not match real-life experiences regarding the reasons cited against webcam use. Thus, whether or not parents had used a webcam seemed to influence their attitudes towards the system.

However, with regard to factors for webcam use, the results illustrated an intersection of experiences and expectations. Also, most parents would choose to use a webcam despite alluding to their possible risks.

Concerning the factors against webcam use, we found that expectations and experiences mainly differentiate. This differentiation was marked when comparing webcam experience and expectations with regard to the control function of webcams. We found that parents without webcam experience feared an increase in mental stress and thus higher anxiety levels. Conversely, this point was of little importance for parents with webcam experience. Current scientific literature shows diverse findings. For example, Kubicka et al. [20] and Rhoads et al. [15] described decreased anxiety and parental stress through webcam use in NICUs. Kerr et al. [11] have elicited that parental well-being appeared to improve when parents had assurance about their child’s health. Well-being and maternal recovery in the postnatal stage were found to be linked in a study by Brown and Lumley [21]. By contrast, Kerr et al. [11] and Le Bris et al. [12] also reported that webcam use could increase tension in few parents. The discrepancy among parents could be attributable to the variety of their experiences in the strenuous postnatal stage. The imagination of an additional stimulus, such as the possibility of a webcam’s presence, can be perceived as a further stressor in already onerous circumstances.

The increase in feelings of perceived emotional closeness within both parental groups was another main aspect of our analysis. The results were in line with findings from previous studies [11, 12]. Emotional proximity has been described as essential for the well-being of a child; it also influences the affective relationship between parent and child [22]. However, our analysis showed that although webcam use was considered convenient for bridging physical distance, it was also thought to risk becoming a permanent substitute for parental visits. This view was held by parents without webcam experience. By contrast, Hawkes et al. [13] found that most parents with webcam experience did not believe that webcam use would influence parental visiting behaviour. This finding is consistent with those of Yeo et al. [23] and Kirolos et al. [24], who reported that webcam use or video updates did not affect the number of parental visits. Although these effects have not been investigated systematically, it can be questioned whether the parental desire to be physically close to and bond with the child is affected by webcam use. It remains unclear whether this concern might be valid for parents who make actual use of webcams in the NICU setting.

While parents with webcam experience did not perceive serious concerns regarding their relationship with health care professionals, parents without webcam experience expected this to be an issue. Le Bris et al. [12] explored a likely reason for this perception. Webcam presence was perceived as harmful for the relationship between health care professionals and parents because parents feared the reduced presence of professionals. Parents also feared that health care professionals might experience webcam-associated anxiety that might increase the probability of medical errors.

To mitigate these risks for the relationship between parents and health care professionals, the following options may be considered: a deactivation-switch and regulated hours. A switch that enables caregivers to turn off the webcam during medical care routines can reduce parents’ compulsion to monitor the quality of care. Regulated hours for virtual visits could reduce the parental urge to control the clinicians’ presence Also, these options could be considered reasonable for health care professionals by enabling them to decide when the webcam is activated. This participatory approach could reduce feelings of control and surveillance, which could positively impact the relationship between health care professionals and parents.

As shown in our analysis, both parent groups believed that webcam use can positively influence breast milk expression. These findings are in accordance with the work of Kerr et al. [11] and Weber et al. [14]. Furthermore, breast milk expression is perceived to enhance satisfaction regarding the maternal role in infant care [25], which is a motivating factor during the strenuous postnatal stage [26]. Mothers also perceived the nourishing of their child as a possibility to connect [27] and foster emotional closeness [28].

Our analysis showed that privacy concerns were important for both parent groups. Le Bris et al. [12] also discussed the relevance of privacy protection. Modern technologies, including webcams in NICUs, can entail a loss of privacy for users and especially for children if their parents do not exercise prudence. With regard to webcam use, the right to privacy applies especially regarding the publication of moving or still images. It is crucial for parents to understand that webcam use is intended for private use only and pictures must not be published online. Before implementation, it is indispensable for decision-makers to consider the legal and ethical aspects concerning privacy and data risks. Security measures and the confidentially of data are noteworthy concerns given the risk of cybersecurity and hacking activities in hospitals [29].

The technology explored in our study showed potential value for helping parents to navigate through their neonatal journey. Analysis revealed that the need to use the technology does exist, especially under the premise of parental mental health. In their qualitative review, Al Maghaireh et al. [30] ascertained that the child’s absence was one major cause for parents’ poor mental well-being. Other studies have emphasised the need to support emotional closeness between parent and child in the NICU setting [31]. Melnyk et al. [32] similarly suggested that a support of parent–infant interaction can decrease postnatal depression in parents of preterm infants. Hence, the connection between parent and infant appears essential for parental well-being. Implementing webcam devices in standard care, presents a way to meet parental demands and limit the negative effects of a premature birth on parents’ mental health.

However, the emotional closeness that webcam use fosters does not substitute for the physical presence of parents. Webcams function only as a tool to mitigate the problem of parent–child separation. It is still crucial to bring physical closeness between parents and children to the fore. Therefore, promotion of family-centred care and rooming-in practices for parents of VLBW children should be prioritised [33]. A prime example appears in the study by Ortenstrand et al. [34], where the provision of family facilities to stay in the NICU reduced the infants’ length of stay in the unit. Similar applications, such as asynchronous video messaging [24] or a short message service for medical updating [35] may present alternatives to webcam use, and could similarly mitigate the negative consequences of webcam use.

### Strengths and limitations

Several limitations might have influenced the results in this study. First, the sample included more mothers than fathers. Few fathers were available for interview participation and their perspectives might therefore be under-represented in this analysis.

Second, it can be assumed that parents who decided to use a webcam system might be more approving of the system than those who had never had the opportunity to experience webcam use. It was beyond the scope of our study to analyse the views of parents who had rejected webcam use in the NICU setting.

Third, some parents were interviewed when their children were no longer hospitalised. The perception of parents with hospitalised children might deviate from those whose children’s NICU stay lies in the past. Although this enabled us to capture the parents’ perceptions in various conditions, the content might have differed if all parents had been interviewed during their NICU stay.

The aim of qualitative research is not to make generalizable statements but rather to investigate and describe realities and to explore interactions. Therefore it is not necessary to achieve a large sample size in order to sufficiently address a research problem [36]. Nonetheless, our sample is appropriate concerning sample size and heterogeneity for the research purpose of contrasting experiences and expectations towards webcam use; hence, the results hold scientific value. The investigator triangulation also allowed various research perspectives and increased the credibility of data.

Results need to be viewed with the awareness that the perception of webcam use depends on parents’ individual patterns. Contextual factors – such as family and work, the availability of mobile devices, distance to the ward, the parent’s own health condition, and the attitude to technology – strongly influence parents’ perceptions about webcams in the NICU setting. These factors differed too much thematically to be included as categories in the analysis.

Notwithstanding these limitations, this study offers insight into parents’ opinions concerning webcam use. The analysis contrasted their expectations and actual experiences. This contrast, has not been explored until now. In addition, we conducted a relatively large number of interviews, which is another strength of the study.

## Conclusion

As the present study illustrates, parental expectations and experiences regarding webcam use are generally positive. Overall, the anxieties of parents who lacked webcam experience were not experienced by parents who had actually used a webcam. Nevertheless, these fears exist and must be addressed when considering webcam implementation. Furthermore, webcam implementation is associated with effort and costs; hence, decision-makers should assess the utility value that webcams can provide for parents. The results of this study can provide a foundation for this task.

It is necessary to emphasise the positive outcomes of webcam use. Although parents were partly critical of the system, the main conclusion is that webcam use presents a way to reduce the consequences of separation between parents and their children. It mentally relieves parents in the postnatal phase. It is of potential value for parents’ well-being and can ease the challenges of a preterm birth.

More importantly, the findings indicate the relevance of a precise explanation of the system to the parents. Expectations and actual experiences were inconsistent and depended on individual and contextual factors; hence, decision-makers should address individual needs by educating parents about the potential risks and benefits that webcam use can signify. A possible way to support parents who intend to use a webcam could be a parents’ manual for webcam use.

Given the increasing relevance of e-health interventions and the visitation regulations during the SARS-CoV-2 pandemic, further consideration should be given to the implementation of webcams. There is scope for further research to determine the webcam’s benefits. An upcoming quantitative evaluation as part of the Neo-CamCare project will attempt to close some of the research gaps.

## Data Availability

The data used and analysed during the current study are available from the corresponding author on reasonable request.

## Abbreviations

NICU: Neonatological Intensive Care Units
VLBW: Very Low Birth Weight
IMVR: Institute for Medical Sociology, Health Services Research, and Rehabilitation Science
